# Explainable Artificial Intelligence to Investigate the Contribution of Design Variables to the Static Characteristics of Bistable Composite Laminates

**DOI:** 10.3390/ma16155381

**Published:** 2023-07-31

**Authors:** Saeid Saberi, Hamid Nasiri, Omid Ghorbani, Michael I. Friswell, Saullo G. P. Castro

**Affiliations:** 1 Department of Mechanical Engineering, Isfahan University of Technology, Isfahan 84156-83111, Iran; s.saberi@alumni.iut.ac.ir; 2 Department of Computer Engineering, Amirkabir University of Technology (Tehran Polytechnic), Tehran 159163-4311, Iran; h.nasiri@aut.ac.ir; 3 Department of Engineering, Kharazmi University, Tehran 15719-14911, Iran; std_omidghorbani2001@khu.ac.ir; 4 College of Engineering, Swansea University, Bay Campus, Swansea SA1 8EN, UK; 5 Department of Aerospace Structures and Materials, Delft University of Technology, Kluyverweg 1, 2629HS Delft, The Netherlands

**Keywords:** composite, bistable, artificial intelligence, machine learning, snap-through, correlation, SHAP, XGBoost

## Abstract

Material properties, geometrical dimensions, and environmental conditions can greatly influence the characteristics of bistable composite laminates. In the current work, to understand how each input feature contributes to the curvatures of the stable equilibrium shapes of bistable laminates and the snap-through force to change these configurations, the correlation between these inputs and outputs is studied using a novel explainable artificial intelligence (XAI) approach called SHapley Additive exPlanations (SHAP). SHAP is employed to explain the contribution and importance of the features influencing the curvatures and the snap-through force since XAI models change the data into a form that is more convenient for users to understand and interpret. The principle of minimum energy and the Rayleigh–Ritz method is applied to obtain the responses of the bistable laminates used as the input datasets in SHAP. SHAP effectively evaluates the importance of the input variables to the parameters. The results show that the transverse thermal expansion coefficient and moisture variation have the most impact on the model’s output for the transverse curvatures and snap-through force. The eXtreme Gradient Boosting (XGBoost) and Finite Element (FM) methods are also employed to identify the feature importance and validate the theoretical approach, respectively.

## 1. Introduction

**Figure 1:**
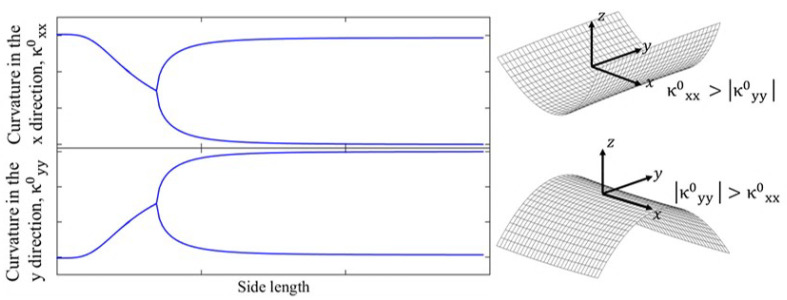
Change in transverse curvatures with side length for cross-ply bistable composite laminates.

Bistable composite laminates not only exhibit two stable shapes but can also switch their shapes between these states in response to appropriate loads. These outstanding features lead to their employment in a range of applications, including energy harvesting [1,2], soft robots [3], and other structures [4,5,6,7,8,9]. The bistability of unsymmetric laminates is a result of the residual thermal stresses generated from the inequality of the thermal expansion coefficients in each ply which are produced during the curing process [10]. Cross-ply bistable laminates have two stable cylindrical shapes; in each shape there are two transverse curvatures, which are the most important characteristics to control the cured shapes of these structures [11]. The variation in the two transverse curvatures with the side length of a cross-ply bistable laminate is illustrated in Figure 1.

For lengths less than the critical length at the bifurcation point, the plate has just one unstable shape. After this point, two stable cylindrical configurations emerge, which have opposite geometries.

**Figure 2:**
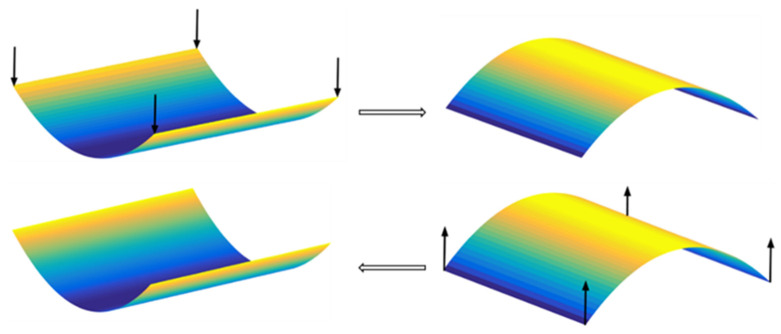
Snap-through between two stable configurations of a bistable laminate fixed at the centre and subjected to four equal concentrated forces at the corners.

One of the interesting properties of bistable and multi-stable composite structures is the snap-through behaviour between the stable configurations. Thus, their shape can be changed from one stable equilibrium shape to the other by applying a sufficiently large external force, which is called snap-through. This jumping between two stable configurations is strongly nonlinear and there is a sudden mechanism, such that the structure experiences large amplitude displacements [12]. The mechanism to change the shape for a bistable laminate fixed at the centre and subjected to four equal concentrated forces at the corners is shown in Figure 2.

Like other composite structures, the material properties, geometry, and environmental conditions may have significant impacts on the curvatures and snap-through force of bistable composites [13]. Hence, utilizing these composites in real-world structures requires the development of our knowledge of the exact influences of these conditions on the desired behaviours.

A range of research has been carried out on the static behaviours of bistable composites. It was proved that thin unsymmetric laminates subject to thermal load can give two stable configurations, in addition to the unstable saddle shape, which classical lamination theory could not predict [14]. Hyer [15] developed an analytical approach based on Rayleigh–Ritz minimisation of the total potential energy using assumed displacement functions, which could determine the two stable cylindrical shapes. Using this method, Dano and Hyer [16] studied the cured shapes of bistable composites for cross-ply and angle-ply laminates and investigated the effect of the stacking sequence on the curvatures. Because of the types of the displacement fields that they used, the curvatures were predicted to be constant along the edges of the laminates, while, in reality, these curvatures vary. Cho and Roh [17] presented a simplified higher-order plate theory for laminates with linear transverse shear profiles by applying Rayleigh–Ritz approximation and considering the slippage effects. The curvatures of bistable laminates predicted using this model were more accurate compared to the previous method [16]. Cantera et al. [18] investigated analytically and experimentally the impacts of the geometric properties and length-to-width ratio on the curvatures and out-of-plane displacements of cross-ply bistable composites by considering the hygrothermal effects. They showed that increasing the aspect ratio leads to increased curvatures. Brampton et al. [19] investigated the effect of uncertainty in the input variables on the major curvature of bistable laminates by considering the temperature dependency of material properties. They showed that the major curvature has the highest sensitivity to uncertainty in the Young’s moduli and thermal expansion coefficients. The sensitivity of the stable shapes to each parameter was examined by defining a ±5% change in the inputs using the Rayleigh–Ritz method and the total potential energy. Zhang et al. [20] analysed the curvatures of bistable composite shells under thermo-mechanical loading through theoretical and finite element methods by considering the material properties as functions of temperature. The results indicated that the through-thickness thermal gradients and uniform temperature field have a substantial effect on the second stable curvature. Cantera et al. [21] modelled the non-uniform curvatures of [0/90] bistable composite plates subject to thermal loads and demonstrated that increasing the length-to-thickness ratio causes a reduction in the cylindrical curvatures. Wu et al. [22] built an analytical model for shell structures considering the hydrothermal effect and analysed the influences of moisture and heat on the bistability of cylindrical shell laminates. Regarding their results, with rising humidity and temperature, the twisting curvature increases. Chillara et al. [23] introduced an approach to develop non-cylindrical curved shapes for mechanically prestressed laminates based on strain energy minimisation. Chai et al. [24] examined the effect of hygrothermal conditions on bistable anti-symmetric composite shells through the principle of minimum potential energy. They proved that increasing both moisture and temperature can enhance the principle and twisting curvatures in angle-ply bistable shells. Zhang et al. [25] designed a new bistable hybrid laminate embedded with some bimetallic strips and examined the impacts of the thickness, width, and material properties of these strips on the principal curvature of the laminate by developing an analytical model based on Dano’s theory. Saberi et al. [11] analysed the bistability probability of five types of bistable laminates using a combination of the principle of minimum potential energy and the Rayleigh–Ritz and subset simulation methods. They indicated that the thermal and moisture expansion coefficients have the highest influences on the region of bistability.

In the case of the snap-through phenomenon, different aspects of the snap-through process for bistable composite laminates under external forces and moments have been studied via experiment, finite element modelling, and theoretical methods by a number of researchers. Dano and Hyer [12] studied the snap-through of three types of unsymmetric bistable laminates with different stacking sequences using the Rayleigh–Ritz technique and virtual work and also obtained the snap-through force theoretically and experimentally. Mattioni et al. [26] numerically estimated the equilibrium configurations and snap-through behaviour of multi-stable composites using finite element analysis and estimated the critical temperature and load to induce bifurcation. Pirrera et al. [27] used a Ritz model with high-order polynomials and path-following algorithms to study the effects of important parameters such as panel geometry, temperature variation, and various lay-ups on the stable configurations and snap-through load of bistable cylindrical panels. Cantera et al. [28] proposed a model to analyse the snap-through and snap-back in bistable cross-ply laminates supported at four points and actuated by a concentrated load based on the Rayleigh–Ritz method and the minimisation of total potential energy. Emam [29] investigated the snap-through and free vibration behaviour of bistable cross-ply composite laminates using a simplified Rayleigh–Ritz model with four time-dependent parameters for the general dynamic response. Zhang et al. [30] theoretically and experimentally investigated the dynamic snap-through phenomena of a square bistable laminate composite panel under foundation excitation. They showed that the geometric dimensions and the numbers of layers can change the critical point for the snap-through. Pan et al. [31] analysed the snap-through response of bistable hybrid symmetric laminates with cantilever boundary conditions using the commercial software Abaqus, and these results were verified by experiments. The results indicate that the lay-up has a notable impact on the snap-through response. Zhang et al. [32] examined experimentally the bistable behaviour of an anti-symmetric laminated cylindrical shell at different temperatures. The results demonstrate that increasing the temperature leads to a decrease in the snap-through load.

In recent years, many researchers have used the machine learning method to study the behaviour of bistable composites. Chau et al. [33] examined the optimisation process for designing bistable composites by employing a hybrid model, which includes an adaptive neuro-fuzzy inference system (ANFIS), statistical methods, fuzzy logic algorithms, finite elements, a desirability function strategy, and lightning attachment procedure optimisation (LAPO). In their study, Liu et al. [34] created a machine learning algorithm for the purpose of designing and optimizing a curved beam with bistable properties. Their model accurately predicted the nonlinear behaviour of the beam and was used to optimise the structure according to different objectives, including stiffness and forward- and backward-snapping forces.

There has been considerable research on the behaviour of bistable composites. However, it is still necessary to expand our horizon on the exact correlations between the input variables and curvatures of the stable configurations because curvatures are the most significant factor to design bistable structures with a desired deformation. Previous papers only focus on calculating the snap-through forces and examining the mechanisms. Therefore, we need to investigate the effect of each of the design parameters on this load, and this work fills this gap. Although [19] and [11] studied the sensitivity of the major curvature and the bistability region of bistable laminates, respectively, this contribution aims to gain a deeper insight into the importance of the input features of bistable composite laminates in a new way so that it not only evaluates relationship magnitudes among design variables and correlates them with the transvers curvatures but also ranks them on the basis of their effect on the outputs. Another novelty of this work is associated with unveiling the influences of input variables on the snap-through load. In addition, the approach used here can be generalised to other multi-stable and complex composite structures, in which it is not possible to analysis them theoretically. As a result, in the present research, an analysis is conducted to determine the exact influences of input variables, such as geometry features, material properties, environmental temperature, and moisture, on the curvatures of the stable shapes and the load required to change these shapes using explainable machine learning. A combined method using the principle of minimum potential energy, Rayleigh–Ritz, and SHAP is employed. Firstly, the equations of bistable composite laminates are derived through the principle of minimizing the potential energy and the Rayleigh–Ritz method. After determining the curvatures of the stable configurations and the snap-through loads, the effect of the variables on these outputs is investigated through SHAP. Shapley values have recently become popular since they offer better interpretability and enhance the system’s trustworthiness and transparency to convert any complex black-box machine learning into a rational human basis system. The results associated with the importance of features and their correlations are also obtained using XGBoost and the Pearson correlation method.

## 2. Methodology

### 2.1. Theoretical Model of Bistable Laminates

To derive the equations, the principle of minimum potential energy is employed. The total potential energy for composite laminates is:

(1) $$\Pi =\int_{-L_{y}/2}^{L_{y}/2}\int_{-L_{x}/2}^{L_{x}/2}\int_{-h/2}^{h/2}{\frac{1}{2}\sigma }^{T}\varepsilon dz\;dxdy$$

where *h*, $L_{x}$, and $L_{y}$ denote the thickness and side lengths of the laminate, and σ and ε are the stress and strain tensors, respectively [35]. Regarding classical lamination theory, the stress–strain relation for the *k*th layer by considering the thermal and moisture conditions can be written as:

(2) $$\sigma ={\bar{Q}}^{k}\left(\varepsilon -\varepsilon_{t}-\varepsilon_{m}\right)$$

where ${\bar{Q}}^{k}\;$is the transformed reduced stiffness of the *k*th layer. $\varepsilon_{t}$ and $\varepsilon_{m}$ are the thermal and moisture strains expressed as $\varepsilon_{t}=\alpha ∆T$ and $\varepsilon_{m}=\beta ∆C$, where $\alpha_{\;}={\left[\begin{array}{ccc} \alpha_{x} & \alpha_{y} & \alpha_{xy} \end{array}\right]}^{T}$ and $\beta_{\;}={\left[\begin{array}{ccc} \beta_{x} & \beta_{y} & \beta_{xy} \end{array}\right]}^{T}$ are the vectors of thermal and moisture expansion coefficients, respectively, ∆T is the temperature variation, and ∆C is the moisture variation.

ε is the total strain field that should include the Von Karman strain terms because of the nonlinear behaviour of bistable laminates. $\varepsilon =\varepsilon^{0}+{z\kappa }^{0}$, where $\varepsilon^{0}\;$and $\kappa^{0}$ are the strain and curvature vectors at the mid-plane expressed as:

(3) $$\varepsilon^{0}=\left[\begin{array}{c} {\varepsilon^{0}}_{xx}\\ {\varepsilon^{0}}_{yy}\\ {\varepsilon^{0}}_{xy} \end{array}\right]=\left[\begin{array}{c} \frac{\partial U_{0}}{\partial x}+\frac{1}{2}{\left(\frac{\partial W_{0}}{\partial x}\right)}^{2}\\ \frac{\partial V_{0}}{\partial y}+\frac{1}{2}{\left(\frac{\partial W_{0}}{\partial y}\right)}^{2}\\ \frac{1}{2}\left(\frac{\partial U_{0}}{\partial y}+\frac{\partial V_{0}}{\partial x}+\frac{\partial W_{0}}{\partial x}\frac{\partial W_{0}}{\partial y}\right) \end{array}\right]\;\kappa^{0}=\left[\begin{array}{c} {\kappa^{0}}_{xx}\\ {\kappa^{0}}_{yy}\\ {\kappa^{0}}_{xy} \end{array}\right]=\left[\begin{array}{c} -\frac{\partial^{2}W_{0}}{\partial x^{2}}\\ -\frac{\partial^{2}W_{0}}{\partial y^{2}}\\ -2\frac{\partial^{2}W_{0}}{\partial x\partial y} \end{array}\right]$$

where $U_{0},{\;V}_{0},and\;W_{0}\;$are the in-plane and the out-of-plane displacements in the *x*, *y,* and *z*, directions, respectively. After substituting Equations (2) and (3) into Equation (1), the total potential energy is given by:

(4) $$\Pi =\int_{-L_{y}/2}^{L_{y}/2}\int_{-L_{x}/2}^{L_{x}/2}(\frac{1}{2}{\left[\begin{array}{c} \varepsilon^{0}\\ \kappa^{0} \end{array}\right]}^{T}\left[\begin{array}{cc} A & B\\ B & D \end{array}\right]\left[\begin{array}{c} \varepsilon^{0}\\ \kappa^{0} \end{array}\right]-{\left[\begin{array}{c} N^{t}\\ M^{t} \end{array}\right]}^{T}\left[\begin{array}{c} \varepsilon^{0}\\ \kappa^{0} \end{array}\right]-{\left[\begin{array}{c} N^{m}\\ M^{m} \end{array}\right]}^{T}\left[\begin{array}{c} \varepsilon^{0}\\ \kappa^{0} \end{array}\right])dx\;dy$$

where *A*, *B*, and *D* indicate the in-plane, coupling, and bending stiffness matrices, respectively, given by:

(5) $$A_{ij\;}=\sum_{k=1}^{a}{\bar{Q}}_{ij}^{\left(k\right)}\left(z_{k}-z_{k-1}\right)\;B_{ij\;}=\frac{1}{2}\sum_{k=1}^{a}{\bar{Q}}_{ij}^{\left(k\right)}\left({z_{k}}^{2}-{z_{k-1}}^{2}\right)\;D_{ij\;}=\frac{1}{3}\sum_{k=1}^{a}{\bar{Q}}_{ij}^{\left(k\right)}\left({z_{k}}^{3}-{z_{k-1}}^{3}\right)$$

where the index *k* indicates the different plies in the laminate and *a* is the number of plies (in this work, *k* = 1, 2 and *a* = 2). Also, ${{\bar{Q}}_{\;}^{\;}}^{k}$ is the transformed reduced stiffness of the *k*th layer. $N^{t,m}$ and $M^{t,m}$ denote the hydrothermal stresses and moment resultants, respectively, given as:

(6) $$N_{i}^{t}=\sum_{k=1}^{a}\sum_{j=1}^{b}{\bar{Q}}_{ij}^{\left(k\right)}\alpha_{j}^{\left(k\right)}∆T\left(z_{k}-z_{k-1}\right)\;\;\;M_{i}^{t}=\frac{1}{2}\sum_{k=1}^{a}\sum_{j=1}^{b}{\bar{Q}}_{ij}^{\left(k\right)}\alpha_{j}^{\left(k\right)}∆T\left({z_{k}}^{2}-{z_{k-1}}^{2}\right)\;N_{i}^{m}=\sum_{k=1}^{a}\sum_{j=1}^{b}{\bar{Q}}_{ij}^{\left(k\right)}\beta_{j}^{\left(k\right)}∆C\left(z_{k}-z_{k-1}\right)\;M_{i}^{m}=\frac{1}{2}\sum_{k=1}^{a}\sum_{j=1}^{b}{\bar{Q}}_{ij}^{\left(k\right)}\beta_{j}^{\left(k\right)}∆C\left({z_{k}}^{2}-{z_{k-1}}^{2}\right)$$

The plate is considered fixed at the centre and free at the edges, and so the proposed displacement fields in Equation (7) must satisfy these boundary conditions.

(7) $${{\;\;\;U}_{0}}^{\;}\left(x,y\right)=\sum_{i=1}^{m}\sum_{j=1}^{n}t_{ij}x^{i}y^{2(j-1)}\;\;\;\;\;{{\;\;V}_{0}}^{\;}\left(x,y\right)=\sum_{i=1}^{m}\sum_{j=1}^{n}s_{ij}x^{2\left(i-1\right)}y^{j}\;W_{0}\left(x,y\right)=\sum_{i=1}^{m}\sum_{j=1}^{n}r_{ij}{x^{2\left(i-1\right)}y}^{2\left(j-1\right)}$$

where $t_{ij}$, $s_{ij}$, and $r_{ij}$ are the unknown coefficients and $R={\left\{t_{ij},s_{ij},r_{ij}\right\}}^{T}$ is the vector of these unknown coefficients. Because of the boundary conditions, ${\;U}_{0}$ (0,0),$\;{\;V}_{0}$ (0,0), and$\;{\;W}_{0}$ (0,0) are equal to zero. Furthermore, according to Equations (2) and (3), the curvatures are considered variable and not constant, resulting in their estimation with high accuracy compared to considering them constant at the edge of the laminates [11].

By substituting Equation (7) into Equation (4) and integrating, the total potential energy is obtained as a function of the unknown coefficients of the displacement fields. The equilibrium shapes are then obtained by the principle of minimum potential energy so that the variations with respect to the unknown coefficients are determined and the resulting expressions are set to zero [36,37,38]. Thus,

(8) $$\delta \Pi =\frac{\partial \Pi }{\partial r_{ij}}\delta r_{ij}+\frac{\partial \Pi }{\partial s_{ij}}\delta s_{ij}+\frac{\partial \Pi }{\partial t_{ij}}\delta t_{ij}=0$$

This equation may be rewritten as:

(9) $$\frac{\partial \Pi }{\partial r_{ij}}=0,\;\;\;\;\;\;\frac{\partial \Pi }{\partial s_{ij}}=0,\;\frac{\partial \Pi }{\partial t_{ij}}=0$$

Equation (9) is a system of nonlinear equations solved by the Newton–Raphson technique. Three solutions typically exist, two of which are associated with stable equilibria and the third is the unstable equilibrium state [15].

The equilibrium configuration of the bistable plate will be stable if the Jacobian matrix of the potential energy is positive definite, or given mathematically as:

(10) $$\left|\frac{\partial^{2}\Pi }{\partial {R_{i}}^{2}}\right|>0$$

The equilibrium configuration of the plate will be stable if this Jacobian matrix (K (R)) of the potential energy is positive definite.

To estimate the snap-through force for each stable state, we should consider the work done by the external forces. The total potential energy is:

(11) $${\frac{\partial \Pi }{\partial R_{ij}}-W_{f}}^{\;}=0$$

where $W_{f}$ is the work done by the external forces, given by:

(12) $${W_{f}}^{\;}=\sum_{m=1}^{4}f_{m}W_{m}(x,y)$$

where $f_{i}$ is the applied force and$\;W_{i}(x,y)$ is the displacement of the plate at the point where the load is applied. In this research, four equal concentrated forces are applied at the corners of the plate.

### 2.2. SHAP

Some advanced machine learning (ML) methods are considered black box models, and understanding the output of them is very difficult, and interpreting them is crucial [39]. This would be even more important when we consider a more sophisticated model because there is a correlation between model complexity and model interpretability, meaning that it is not so clear how well a complicated model works. This insufficient clarity brings an issue of trustability. This problem can be solved using SHAP because despite the black-box model, in which even the developer cannot explain its specific decisions, the EML methods can interpret results in a way that are understandable to humans [39,40,41]. SHAP is one of the most novel EML methods to study model interpretability [42,43,44,45,46,47,48]. The method has origins in game theory to explain the performance of a machine learning model and evaluate the contribution of each input variable to the model’s output. This method was initially invented for assigning payment to the players according to their contributions towards the total profit [49]. SHAP values can demonstrate the contribution, whether positive or negative, of each input parameter (all records of each parameter together) as well as each record (each singular record of a variable) on the objective and raise the transparency of the prediction model. The Shapley values have also been linked to sensitivity analysis and ranking inputs, providing a good assessment of the existence of dependencies [50,51,52,53,54,55].

To generate an interpretable model, SHAP utilises an additive feature attribution approach, which means it defines the output model as a linear combination of input features (*x*) [46,47]. Indeed, the SHAP explanation model $g\left(x^{\prime }\right)\;$for prediction $f\left(x\right)$ is defined as:

(13) $$f\left(x\right)=g\left(x^{’}\right)=\varphi_{0}+\sum_{i=1}^{P}\varphi_{i}{x^{’}}_{i}$$

where *P* denotes the number of input variables, $\varphi_{0\;}$ is the constant value when there are no features in the model, $\varphi_{i\;}$ denotes the SHAP values, and x′ is a binary parameter that indicates whether the *i*th variable is being observed $\left(x^{\prime }=1\right)$ or not ($x^{\prime }=0$).

There is a single unique solution for Equation (13) which should have three main characteristics, including local accuracy, missingness, and consistency [47]. Local accuracy: when estimating the original model *f* for an input feature *x*, local accuracy requires the explanation of the model *g* for the simplified input *x′* to match the output of *f*. This is satisfied once${\;x=h}_{x\;}(x\prime )$. Missingness: there is no importance for features missing in the input, which means $x^{\prime }=0$ leads to $\varphi_{i}=\;0.$ Consistency: consistency means that altering a feature ($x^{\prime }$) with a large effect cannot reduce the attribution ($\varphi_{i}$) allocated to that feature.

Regarding game theory, the SHAP value for the *i*th input variable is expressed as:

(14) $$\varphi_{i}\left(f,x\;\right)=\sum_{S\subseteq N\backslash \{i\}}\frac{\left|S\right|!\left(P-\left|S\right|-1\right)!\;}{P!}[f_{x}\left(S\cup \left\{i\right\}\right)-\;f_{x}\left(S\right)]$$

where *N* is a vector of features, and *S* is a subset of *N* that represents the number of entries in $x^{\prime }$, which are non-zero. Moreover, $f_{x}\left(S\right)=f\left(h_{x}\left(x^{\prime }\right)\right)=E\left(f\left(x\right)|x_{s}\right)$, where $E\left(f\left(x\right)|x_{s}\right)$ is the value of the function for a subset (*S*) of the inputs. According to Equation (14), the SHAP value for each input variable is obtained, and their importance can be ranked based on their $\varphi_{i}$ values [56].

### 2.3. XGBoost

Extreme gradient boosting [57] is an enhanced and scalable machine learning algorithm based on gradient boosting decision trees. This novel method has been implemented in different engineering fields because of its merits [58,59,60]. First, it can offer high performance for both regression and classification problems utilizing Taylor series expansions of the objective function, efficiently constructing boosted trees and working in parallel. Second, a multi-threading parallel computing model can also be automatically called in XGBoost, which is several times faster than the traditional ensemble learning models [61]. Finally, the regularisation term used in its objective function increases the generalisation ability, making it less likely that the model would be overfitted by data [62,63,64,65,66]. The prediction score of XGBoost is the sum of all the scores in the trees, defined as:

(15) $$\emptyset^{m}=\;\sum_{i=1}^{n}L(y_{i}\;\cdot \;{\hat{\;y}}_{i}^{m})+\sum_{j=1}^{m}\Omega (f_{i})$$

where *n* represents the number of records, *L*() denotes the loss function measuring the error between the target $y_{i}$ and the predicted ${\hat{y}}_{i}^{m}$ to optimise and yield the optimal value, and Ω() is the regularisation term. This regularisation term removes the model complexity to avoid overfitting [67] and is defined as:

(16) $$\hat{Y}=\;\sum_{m=1}^{M}F_{m}(X)$$

where$\;\hat{Y}$ and $F_{m}\;$show a prediction score over all trees and the individual score of each tree in the model, respectively. *M* indicates the number of trees used in the model and ***X*** is the feature vector. The regularised objective function of XGBoost at the *m*th iteration is expressed as:

(17) $$\Omega \left(f\right)=\;\gamma T+\frac{1}{2}\;\lambda \sum_{k=1}^{T}w_{k}^{2}$$

where *T* and *w* denote the number of nodes and the weight of each node, respectively [68]. γ and λ are also two constant values utilised to control the degree of the regularisation.

After building the trees using the training dataset, XGBoost is able to calculate the importance of each input feature based on the weight. In XGBoost, the weight represents the number of times a variable is employed for data splitting and is calculated as:

(18) $${IMP}^{F}=\;\sum_{m=1}^{M}\sum_{l=1}^{L-1}I(F_{m}^{I}\;\cdot \;F)\;I\left(F_{m}^{I}\;\cdot \;F\;\right)\;=\;\left\{\begin{array}{c} 1\;\;\;\;\;\;\;\;\;\mathrm{if}\;\;F_{m}^{I}==F\;\;\;\;\;\;\;\;\;\;\;\;\;\;\;\;\\ 0\;\;\;\;\;\;\mathrm{otherwise}\;\;\;\;\;\;\;\;\;\;\;\;\;\;\;\;\;\;\;\;\; \end{array}\right.\;$$

where *m*, *M*, *L*, and *L* − 1 represent the number of trees, total number of trees, number of nodes in the *m*th tree, and the number of non-leaf nodes of the tree, respectively. Also, $F_{m}^{I}\;$is the feature associated with the node *L*. This model is used to obtain the feature importance of XGBoost to evaluate the feature importance we get from SHAP. In this way, we are able to explain our model and describe which features have the most impact on our prediction.

The randomised search cross validation method was used for hyperparameter optimisation. The obtained parameters employed to build the XGBoost model for this study are listed in Table 1.

### 2.4. The Characteristics of the Laminates

The desired bistable composite plate has an unsymmetric lay-up [0/90] with a 150 mm × 150 mm side length. It should be noticed that material properties are assumed to be independent of temperature. Furthermore, the cure and room temperatures are assumed to be 165 °C and 25 °C, respectively [11]. The coefficient of moisture expansion in the direction of the fibre ($\beta_{11}$) is insignificant and hence assumed to be zero [11]. To predict the contribution features to the curvatures and snap-through load, a number of samples must be considered by defining uncertainty in the inputs using a statistical technique. The mean value and the coefficient of variation in the assumed features are listed in Table 2. The probability distribution of these random parameters is considered to be normal based on goodness-of-fit test results [69,70,71]. By considering these features, 375 sets of the material properties were randomly generated. Then, the equations of the bistable laminates, explained in Section 2.1, were solved for the 375 sets of the materials properties and the desired responses determined. Therefore, in this study, 375 bistable laminates were considered. It should be mentioned that after determining the results for different numbers of samples (200, 300, and 350), no changes in the results occurred when more than 370 samples were considered. From the entire dataset, 60% of samples were randomly used as the training set, 20% as the validation set, and the rest were considered as the test set. The training set was used for model training, while the test set was used for the evaluation of the model. The validation set was used to avoid overfitting and improve the model’s generalisation [72]. Table 3 shows obtained results by XGBoost on the training and test sets.

To validate the theoretical approach, the out-of-plane displacement in the first stable shape were determined using both the analytical method and FEA based on Abaqus (6.14) commercial software package [73]. By considering *m*, *n* = 3 in Equation (7), the corner displacement achieved by the theoretical method is 10.19 mm, while the result obtained by FEM is 10.02 mm. This means that the theoretical method has an acceptable accuracy since the error is less than 1.7%.

To conduct FEA for a bistable composite plate in Abaqus, the following stages should be conducted. After designing the rectangular geometry in the ‘Part’ module, in the ‘Property’ module, the composite lay-ups should be defined according to Table 2. In the next module, to predict the stable configurations, the ‘Static-General’ step is selected to simulate the manufacturing process. It should be notice that ‘Nlgeom’ is considered for the static analysis due to the geometric nonlinearity. Moreover, to obtain reliable results, a damping factor of $10^{-7}$ is defined for the automatic stabilisation. Next, the simulated laminate is meshed using linear quadrilateral (S4R) elements with hourglass control and reduced integration, comprising 1849 elements and 1936 nodes. In the ‘Force’ module, the boundary conditions of the laminate are defined, where the laminate is fixed at the centre. We can simulate the manufacturing process using the ‘Predefined Field’ option in this module by choosing 165 °C and 25 °C for the curing and room temperatures, respectively [5,11].

## 3. Results

### 3.1. Features Importance for the Curvatures

**Figure 3:**
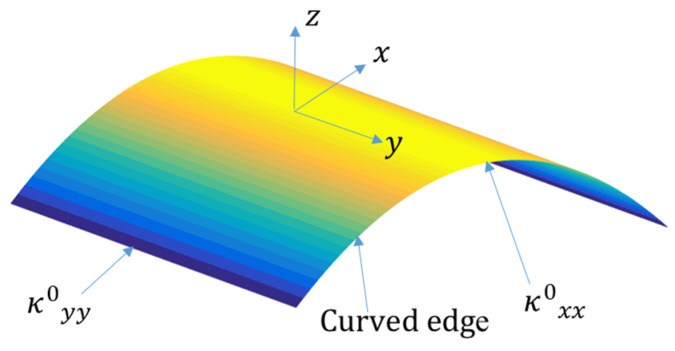
The transverse curvatures for the first stable configuration of the [0/90] bistable laminate.

To determine curvatures of the stable shapes, first, the stable configurations should be predicted using Equation (8), leading to an estimate of the vector *R*. By substituting the displacement fields into Equation (2), the curvatures are calculated. The transverse curvatures of the stable shapes are shown in Figure 3.

**Figure 4:**
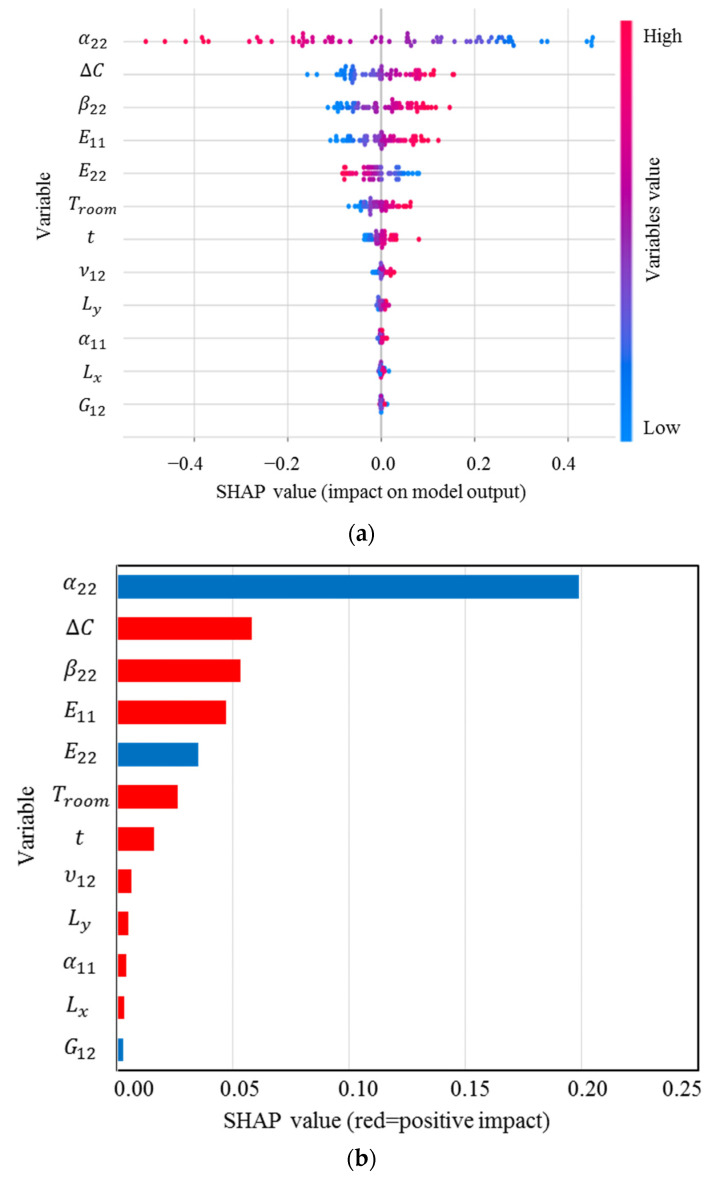
(**a**) SHAP values; (**b**) mean of SHAP values for ${\kappa^{0}}_{xx}$.

Because the transverse curvatures are the most significant characteristic of bistable composites to control their deformed shapes, it is required to identify the precise effect of features on them. SHAP values for ${\kappa^{0}}_{xx}$, the main curvature in the first stable state of the square cross-ply bistable composite plate, are shown in Figure 4. These values rank the input variables based on their importance on the curvature and indicate their correlations.

The distribution and range of impact of the input variables on the curvature can be determined using Figure 4a. Each point on this figure is a Shapley value associated with the input variables and a specific input. The vertical axis from top to bottom displays the order of importance of the input variables, which begins with the most important variable. The horizontal axis shows the SHAP value, and the colour bar indicates the variable’s importance level so that blue to red shows low to high significance. From the SHAP analysis, the most significant input variable in obtaining the curvature is $\alpha_{22}$, which is considerably more influential than the others, followed by ∆C, $\beta_{22}$, $E_{11}$, while $G_{12}$ has the lowest impact on ${\kappa^{0}}_{xx}$ [11,13]. It is clear that $\alpha_{22}$ has a high and negative impact on the output due to the red points on the negative section of the horizontal axis. Figure 4b demonstrates the mean of the SHAP values. The higher the significance factor, the more significant the variable is. Evaluating the magnitude of these relationships reveals that except for $\alpha_{22}$, $E_{22}$, and $G_{12}$ exhibiting a negative correlation with the output, the other input parameters exhibit a positive relationship with ${\kappa^{0}}_{xx}$, meaning increasing $\alpha_{22}$, $E_{22}$, and $G_{12}$ lead to a reduction in this curvature. In summary, this figure would be helpful to design bistable composite structures since it reveals that they have the greatest sensitivity to uncertainties in $\alpha_{22}$.

**Figure 5:**
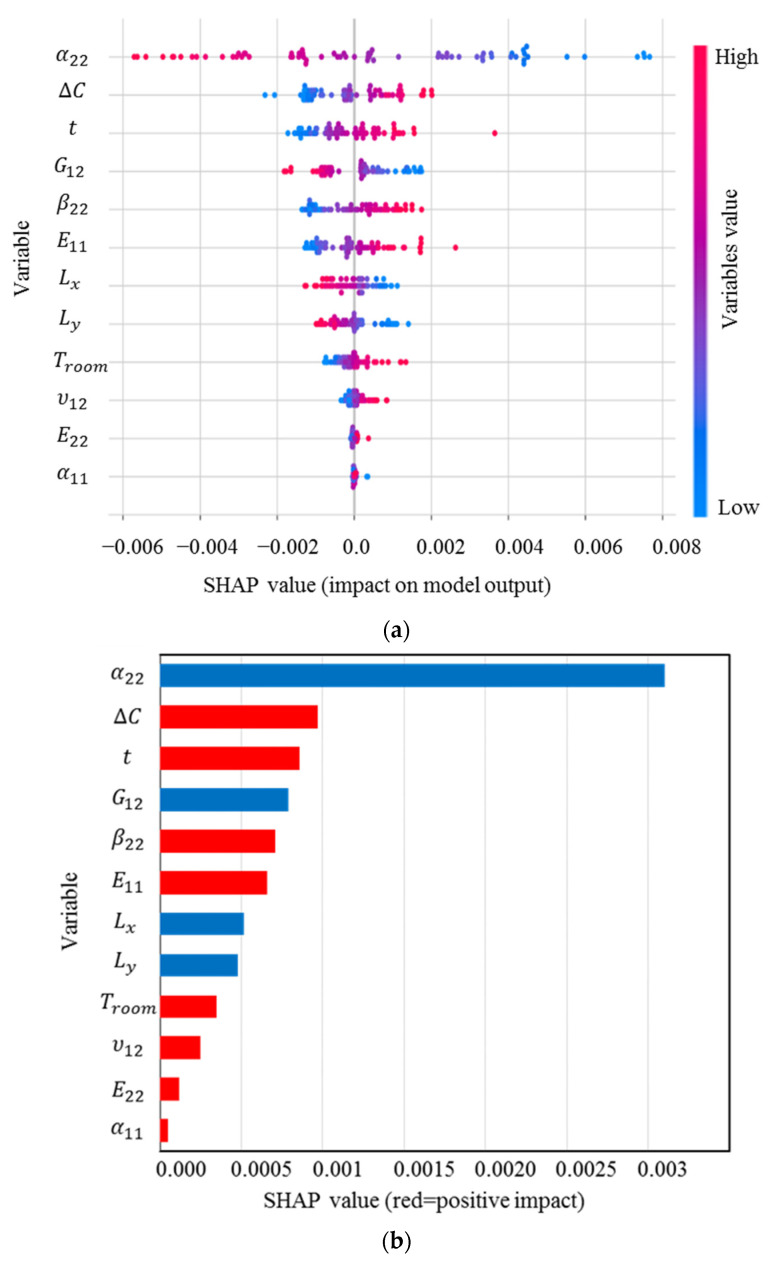
(**a**) SHAP values; (**b**) mean of SHAP values for ${\kappa^{0}}_{yy}$.

The SHAP analysis results for ${\kappa^{0}}_{yy}$ are presented in Figure 5, which shows that this curvature is mostly sensitive to $\alpha_{22}$, and then ∆C, *t*, and $G_{12}$. Interestingly, there is a negative correlation between ${\kappa^{0}}_{yy}$ and $\alpha_{22}$, $G_{12}$, $L_{y}$, and $L_{x}$, i.e., increases in these inputs can reduce the curvature. According to Figure 4 and Figure 5, after manufacturing a cross-ply bistable composite plate, if we want to increase ${\kappa^{0}}_{yy}$ and decrease ${\kappa^{0}}_{xx}$, for example, reducing the length and shear modulus of the laminate can be a good strategy.

**Figure 6:**
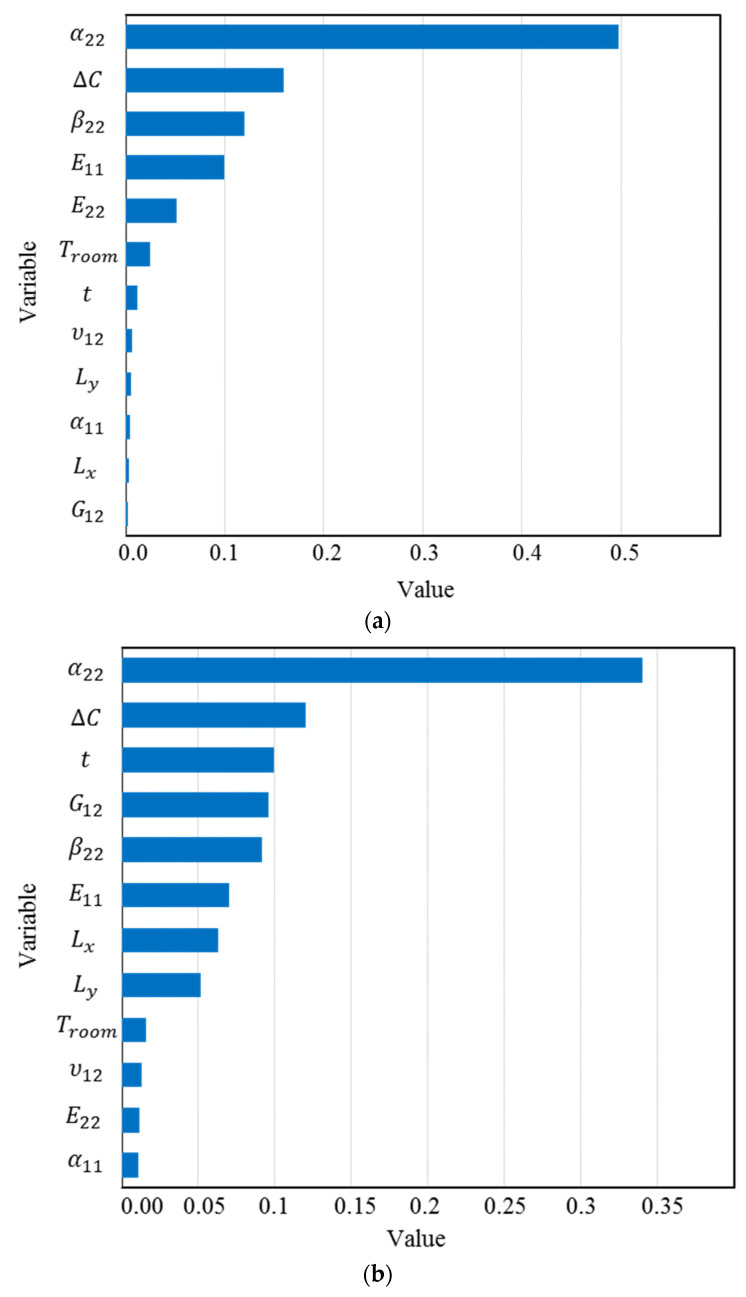
Feature contribution for (**a**) ${\kappa^{0}}_{xx}$ (**b**) ${\kappa^{0}}_{yy}$ determined by XGBoost.

To further strengthen this study, XGBoost, as a novel ML method, is used to determine the feature importance of the input variables. The results of SHAP have a good agreement with the feature importance results obtained from XGBoost, which are presented in Figure 6. Giving this figure, the most important input variables for $\kappa_{xx}$ are $\alpha_{22}$, Δ*C*, and $\beta_{22}$, while for $\kappa_{yy}$, they are $\alpha_{22}$, Δ*C*, and t.

**Figure 7:**
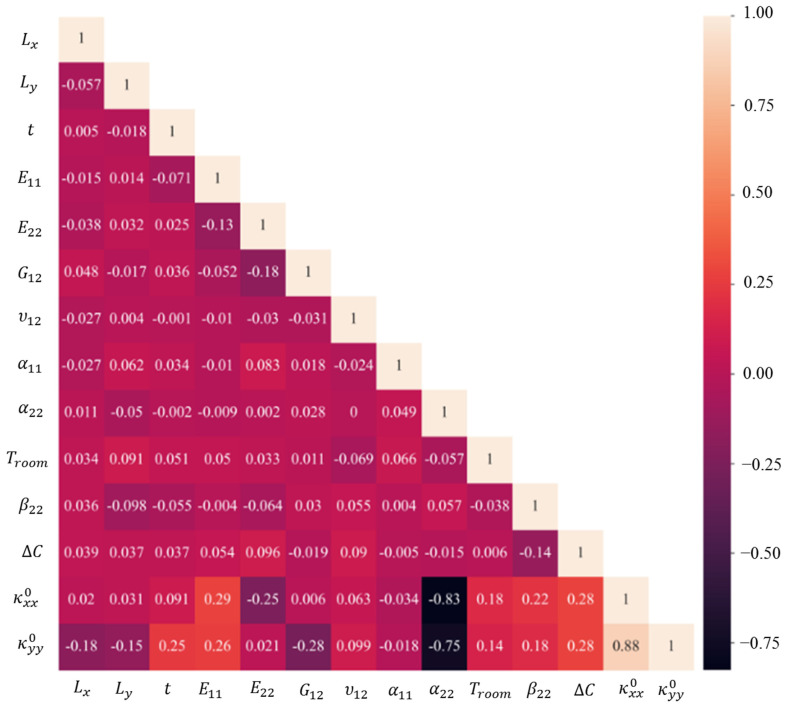
Pearson correlation between the transverse curvatures and input variables.

Pearson correlation is also used to measure the strength of the linear relationship between variables. Note that we can indirectly use Pearson correlation to validate the SHAP results by checking if the variables deemed important by SHAP also have a high correlation with the target variable. However, keep in mind that Pearson correlation only measures the linear correlation between two variables, so it may not capture non-linear relationships between features and the target variable. Figure 7 illustrates the Pearson correlation among these parameters which has a strong agreement with SHAP correlation assessments. For instance, this figure confirms that there is a negative relationship between the curvatures and *α*_22_, and also *α*_22_ has the greatest correlation with them. In the case of the inputs, *E*_22_ and *G*_12_ have the highest correlation.

It should be highlighted that the Pearson correlation only assesses a linear relationship between two variables and plots the impact of variables on the output. However, SHAP evaluates not only linear but also nonlinear multivariable relationships and plots SHAP values for each variable regarding its rank.

**Figure 8:**
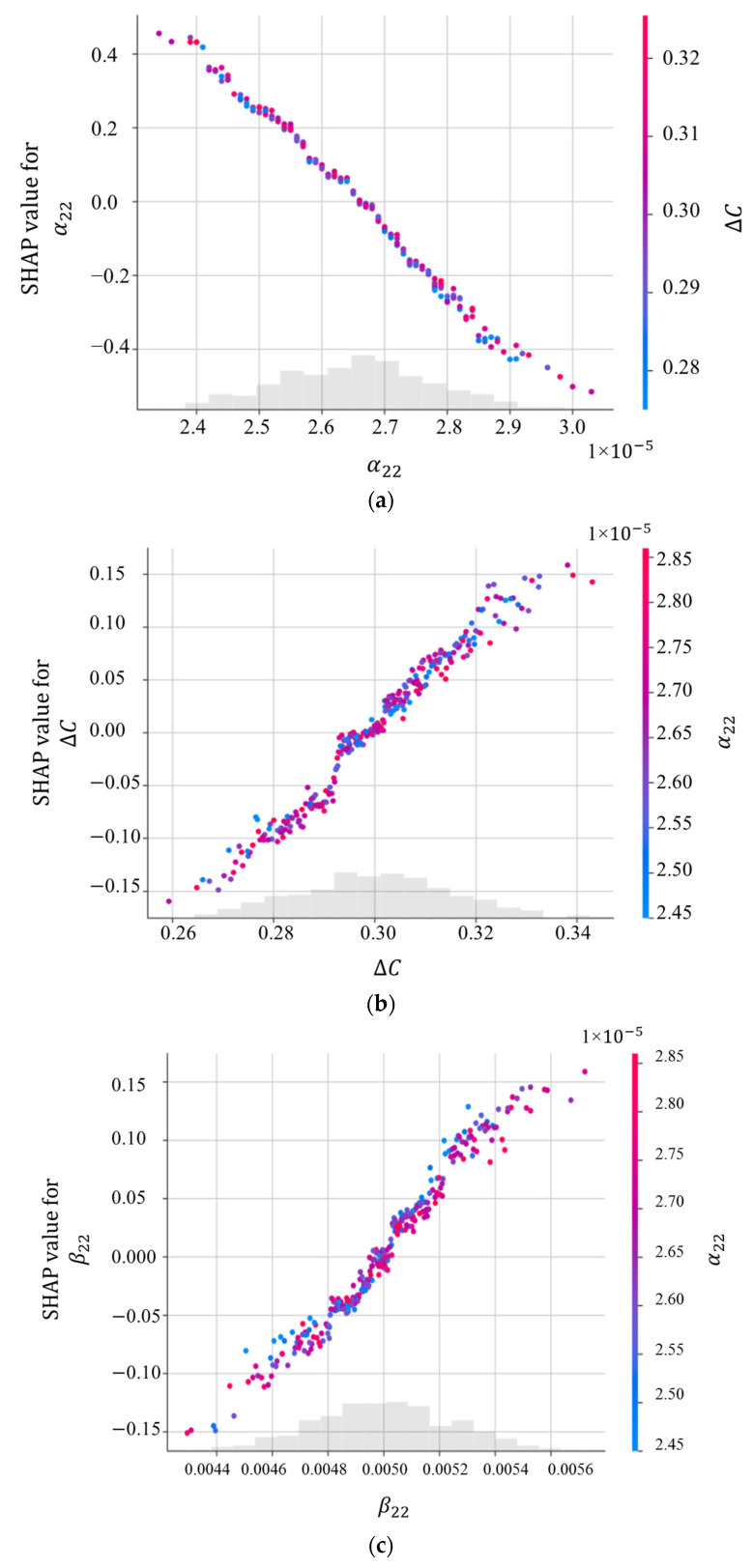

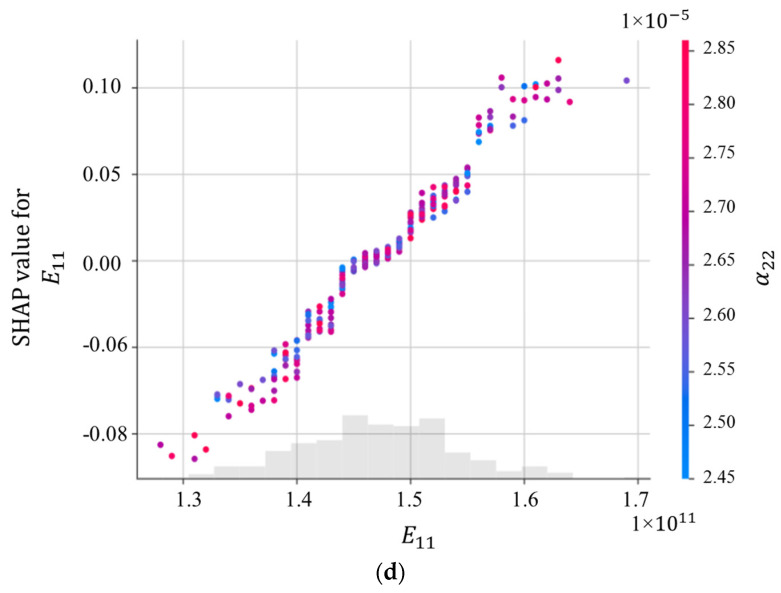
SHAP dependency plots for (**a**) $\alpha_{22}$; (**b**) ∆C; (**c**)$\beta_{22}$; (**d**) $E_{11}$ for ${\kappa^{0}}_{xx}$.

**Figure 9:**
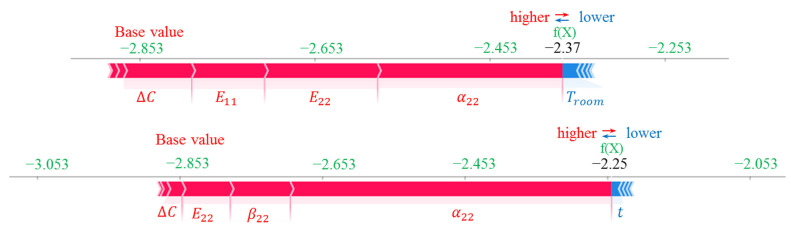
SHAP force plots for two different samples for the prediction of ${\kappa^{0}}_{xx}$.

**Figure 10:**
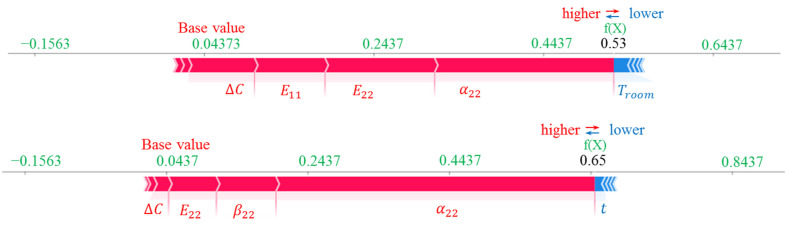
SHAP force plots for two different samples for the prediction of ${\kappa^{0}}_{yy}$.

The SHAP dependency plots, the variation in SHAP values with changes in the input variables, are presented in Figure 8. The SHAP values presented in Figure 4 and Figure 8 are the same, but the latter helps to give a deeper insight into the variation and spread of the SHAP values with the input parameters. According to Figure 8a, there is a relatively linear and negative relationship between $\alpha_{22}$ and ∆C for ${\kappa^{0}}_{xx}$, meaning an increase in $\alpha_{22}$ can reduce its SHAP value. The impact of $\alpha_{22}$ is shown for a variation in ∆C from 0.28 to 0.32. The red values signify high values of ∆C, while the blue represents low values. When $\alpha_{22}$ is more than 2.7 × 10^−5^ (1/°C), the SHAP value for $\alpha_{22}$ is negative. In contrast, there is a quite linear and positive dependency between ∆C and $\alpha_{22}$ (Figure 8b), $\beta_{22}$ and $\alpha_{22}$ (Figure 8c), as well as $E_{11}$ and $\alpha_{22}$ (Figure 8d). Figure 9 and Figure 10 illustrate SHAP force plots for two different samples for the prediction of ${\kappa^{0}}_{xx}$ and ${\kappa^{0}}_{yy}$, respectively. SHAP force plots offer an intuitive way to visualise feature importance and understand local interpretability in machine learning models, enabling better insights into model predictions and aiding in decision-making processes.

### 3.2. Features Importance for the Snap-Through Load

**Figure 11:**
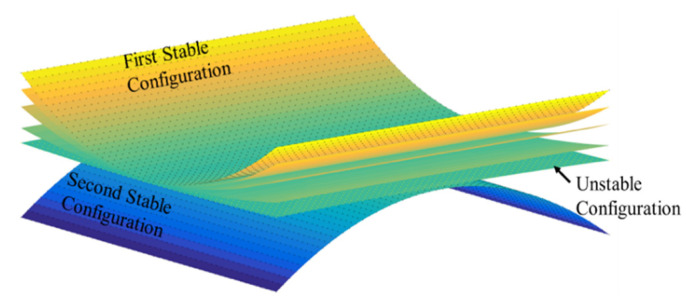
Snap-through process from the first stable state to the second stable state for a bistable composite laminate.

Snap-through phenomenon will occur if a suitable load is applied to bistable laminates and one point of the structure reaches the critical point. The deformation process of a cross-ply bistable laminate during the snap-through from the first stable configuration to the second configuration is demonstrated in Figure 11.

**Figure 12:**
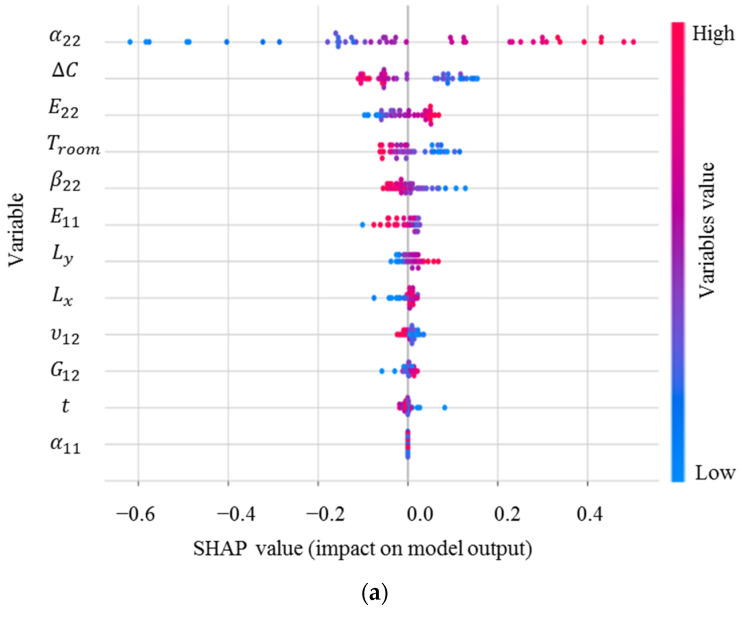

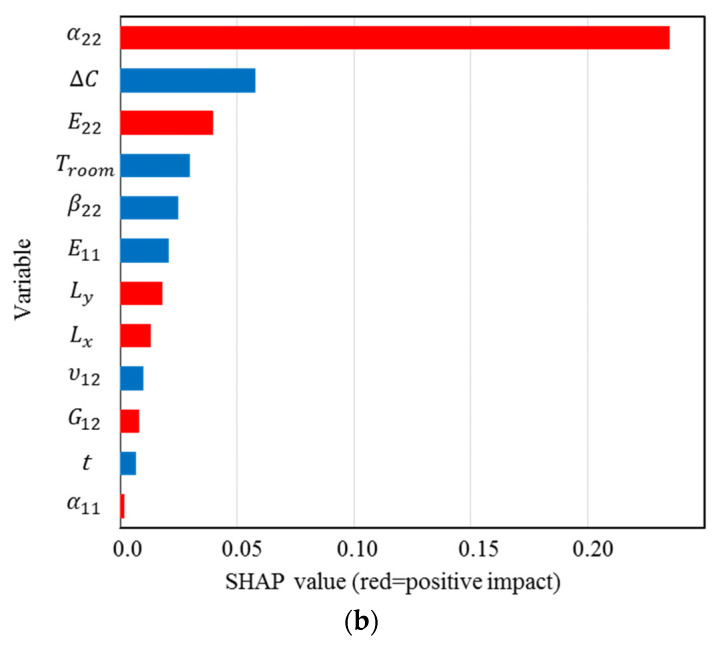
(**a**) SHAP values; (**b**) mean of SHAP values for the snap-through load.

In the following, the effect of these uncertainty sources on the snap-through load will be investigated. The results for the feature importance of the snap-through force obtained using SHAP are shown in Figure 12.

**Figure 13:**
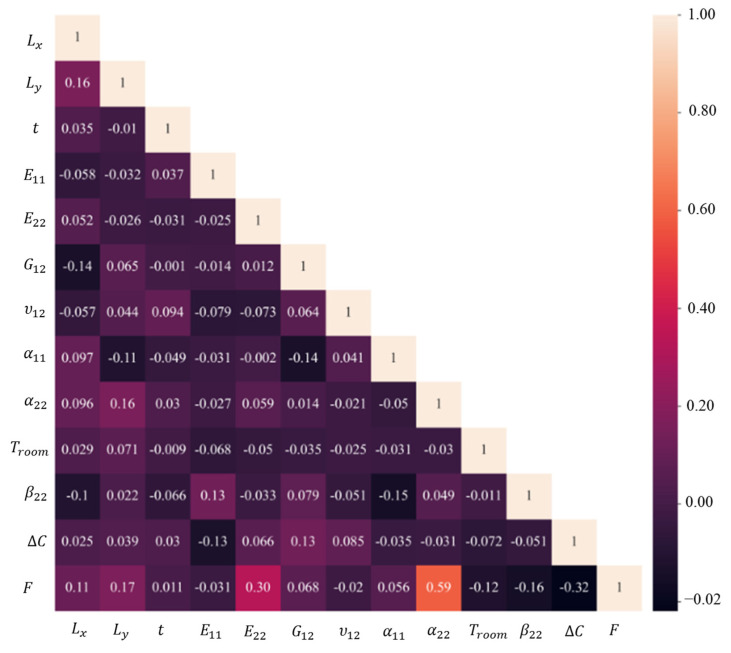
Pearson correlation between the snap-through and input variables.

The most important parameter for the load required to change the configuration of a bistable laminate from the first stable state to second state is the transverse thermal expansion coefficient. After this parameter, ∆C and $E_{22}$ have the highest influence on the output. However, this load has the least sensitivity to the longitudinal thermal expansion coefficient and the thickness. In contrast to the curvatures, $\alpha_{22}$ has a direct relationship with the snap-through force, meaning any increase in this parameter can raise the required load to initiate the jump. Another interesting point is that the difference in moisture saturation (∆C) negatively affects the load. The Pearson correlation among the input variables and the snap-through force is illustrated in Figure 13.

The snap-through force has the highest positive correlation with $\alpha_{22}$, followed by ∆C and $E_{22}$, which is in good agreement with the results of SHAP.

## 4. Conclusions

Engineering problems often perceive some advanced ML techniques as black boxes since humans cannot easily interpret and explain ML predictions. To tackle this barrier and interpret the ML model output, in this work, SHAP, as an XAI approach, was employed. This study investigated the contribution of the design variables, such as ambient moisture and temperature, material properties, and geometric sizes, to the curvatures and snap-through load of bistable composite laminates using novel methods, including SHAP and XGBoost. SHAP effectively evaluated the importance of the input variables in the prediction of output. It was shown that the transverse thermal expansion coefficient, followed by the moisture variation, have the highest effects on the curvatures and snap-through load, while geometric dimensions are generally less important. This research can be generalised to other composite materials and applications to measure the effects of the input variables on outputs, which would be beneficial for reliability analysis and behaviour prediction. The procedure can also be helpful in reducing the computational costs for more complex structures, with a large number of variables. Since the proposed approach can rank the design variables based on their importance, less significant parameters can be recognised and ignored. Although the proposed approach obtained promising results, the small size of the dataset is a limitation of the current study. In future research, we will use a larger dataset and employ other XAI methods, such as LIME, to interpret the model’s output.

## Figures and Tables

**Table 1:** The XGBoost parameters used to build the model.

Parameters	$\mathrm{Value}\;({\kappa^{0}}_{xx})$	$\mathrm{Value}\;({\kappa^{0}}_{yy})$
Base score	0.5	0.5
Booster	gbtree	gbtree
Colsample by level	1	1
Colsample by node	1	1
Colsample by tree	0.7	0.7
Gamma (*γ*)	0	0
Number of estimators (*N*)	900	500
Maximum depth (*D*)	3	3
Reg alpha (*L*_1_)	0	0
Reg lambda (*L*_2_)	1	1
Learning rate (α)	0.05	0.07
Minimum child weight	1	1

**Table 2:** Statistical properties of the random parameters [11].

Property		Mean Value	Coefficient of Variation
Longitudinal elastic modulus	$E_{11}$ [GPa]	146.95	0.05
Transverse elastic modulus	$E_{22}\;[GPa]$	10.702	0.05
Shear module (GPa)	$G_{12}$ [GPa]	6.977	0.05
Poisson ratio	$\nu_{12}$	0.3	0.05
Temperature variation	${T_{room}\;\left[\;{}^{\circ}C\right]}_{\;}$	25	0.05
Longitudinal thermal expansion coefficient	$\alpha_{11}\;\left[1/\;{}^{\circ}C\right]$	5.028 × 10^−7^	0.05
Transverse coefficient of thermal expansion	$\alpha_{22}\;\left[1/\;{}^{\circ}C\right]$	2.65 × 10^−5^	0.05
Moisture expansion coefficient	$\beta_{22}\;$[1/wt%]	0.005	0.5
Moisture variation	ΔC [%]	0.3	0.05
Ply thickness	$t_{ply}\;$[mm]	0.365	0.01
Side length	$L_{x}\;$[m]	0.15	0.01
Side length	$L_{y\;}$[m]	0.15	0.01

**Table 3:** XGBoost’s results on the training and test sets.

Output	Training		Test	
	RMSE	MSE	RMSE	MSE
${\kappa^{0}}_{xx}$	2.30 × 10^−3^	5.40 × 10^−6^	5.10 × 10^−2^	2.60 × 10^−3^
${\kappa^{0}}_{yy}$	3.50 × 10^−4^	1.22 × 10^−7^	1.40 × 10^−3^	2.06 × 10^−6^

## Data Availability

Not applicable.
